# Wireless, Smart Hemostasis Device with All‐Soft Sensing System for Quantitative and Real‐Time Pressure Evaluation

**DOI:** 10.1002/advs.202303418

**Published:** 2023-09-08

**Authors:** Chengjun Zhang, Qing Yang, Xianglin Meng, Haoyu Li, Zexiang Luo, Lin Kai, Jie Liang, Sicheng Chen, Feng Chen

**Affiliations:** ^1^ School of Mechanical Engineering Xi'an Jiaotong University Xi'an 710049 P. R. China; ^2^ Department of Critical Care Medicine The First Affiliated Hospital of Harbin Medical University Harbin 150001 P. R. China; ^3^ State Key Laboratory for Manufacturing System Engineering and Shaanxi Key Laboratory of Photonics Technology for Information School of Electronic Science and Engineering Xi'an Jiaotong University Xi'an 710049 P. R. China; ^4^ School of Electrical and Electronic Engineering Nanyang Technological University 50 Nanyang Avenue Singapore 639798 Singapore

**Keywords:** all‐soft pressure sensors, compact and wireless sensing systems, femtosecond laser, liquid metals, smart hemostasis devices

## Abstract

The properly applied pressure between the skin and hemostasis devices is an essential parameter for preventing bleeding and postoperative complications after a transradial procedure. However, this parameter is usually controlled based on the subjective judgment of doctors, which might cause insufficient hemostatic effect or thrombosis. Here this study develops a compact and wireless sensing system for continuously monitoring the pressure applied on the radial artery and wrist skin in clinical practice. A liquid metal (LM)‐based all‐soft pressure sensor is fabricated to enable conformal attachment between the device and skin even under large deformation conditions. The linear sensitivity of 0.007 kPa^−1^ among the wide pressure range of 0–100 kPa is achieved and the real‐time detection data can be wirelessly transmitted to mobile clients as a reference pressure value. With these devices, detailed pressure data can be collected, analyzed, and stored for medical assistance as well as to improve surgery quality.

## Graphical abstract

This work develops a wireless sensing system upon a hemostasis device. The system consists of liquid metal‐based all‐soft capacitive pressure sensors, tiny reading circuits with low‐power transmission functions, and software‐based model deployment. With these devices, detailed pressure data on retracting operations can be collected, analyzed, and stored for medical assistance as well as improving surgery quality.

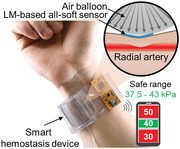

## 1 Introduction

**Figure 1:**
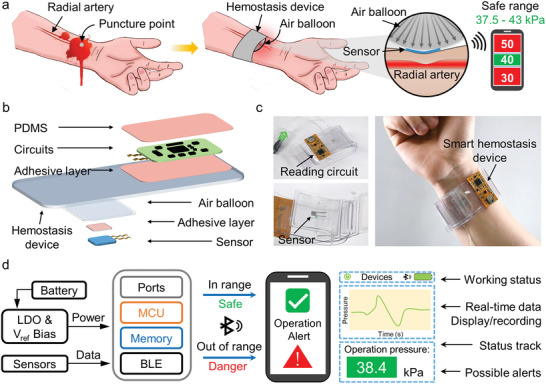
Wireless sensing system upon hemostasis device. a) Schematic view of the smart hemostasis device for subtle surgery. b) Exploded schematic illustration of the active subsystems, enclosure architectures, and customized sensors. c) Images of smart hemostasis device worn on the wrist. d) Block diagram of the system operation with user interface (Note S2, Supporting Information).

The Hemostasis device is a kind of device wrapped around the wrist to prevent massive bleeding after surgery (**Figure** **1**a).^[^ ^1^, ^2^ ^]^ In applications, 13–15 mL of air will be injected into the balloon according to the size of the patient's wrist to compress the radial artery for hemostasis.^[^ ^3^, ^4^ ^]^ In this process, too small applied pressure will lead to bleeding, thereby causing hematoma; while too large pressure will introduce radial artery occlusion and thrombosis. Accurate adjustment of the pressure applied to the radial artery puncture port can improve the hemostatic effect and reduce the morbidity of vascular access complications.^[^ ^5^, ^6^ ^]^ However, due to the individual differences of the patient and the subjective judgment of doctors, the pressure will fluctuate, which will affect the comfort and hemostatic effect of the patient. Therefore, real‐time detection of pressure at the radial artery can effectively contribute to adjust the pressure of air injected into the balloon, so as to achieve accurate attachment, rapid hemostasis, and improve patient comfort, but this has not yet been explored.

Intelligent medical equipment is of great significance for assisting clinical medicine.^[^ ^7^, ^8^, ^9^, ^10^, ^11^ ^]^Although several previous works have been reported on the detection of the pressure applied by bandage or hemostatic devices, they are usually accompanied by the rigid sensing system, which is far from smart and conformal in clinical applications.^[^ ^6^, ^12^, ^13^ ^]^ To solve this dilemma, advanced flexible sensors can be potential candidates to achieve more accurate attachment with skin benefitted by its certain flexibility of the soft matrix, so as to realize precision pressure detection.^[^ ^14^, ^15^, ^16^, ^17^ ^]^ The mainstream pressure sensors include capacitive, resistive, and transistor types.^[^ ^18^, ^19^, ^20^, ^21^, ^22^, ^23^, ^24^ ^]^ Resistive and transistorized pressure sensor usually have a stable detection capability, and would not be affected by surrounding interference, but transistorized pressure sensor is accompanied by the need for continuous power supply and high energy consumption, and the resistive pressure sensor is too sensitive to tensile strain in the application of the deformed devices.^[^ ^22^, ^25^, ^26^ ^]^ Capacitive pressure sensors are widely used in wearable electronic devices due to their high sensitivity to pressure, rapid response, and insensitivity to temperature, and it can be designed to reduce their sensitivity to strain.^[^ ^15^, ^27^ ^]^ Therefore, the flexible capacitive pressure sensors may be the desired option upon the hemostasis device for accurate pressure detection. Although the flexible pressure sensor has been used in medical detection, it is still a great challenge to accurately detect the pressure at the wound, because the balloon will greatly deform when injecting the air, which will lead to the loss of pressure detection capability of the flexible sensor.

Here, we develop a strategy that exploits a wirelessly communicating, compact, and all‐soft sensing system upon a hemostasis device, capable of monitoring the real‐time pressure applied on transradial. The all‐soft pressure sensor, using the gallium‐based liquid metal (GLM) as electrode and double‐side polydimethylsiloxane (PDMS) micropyramids as dielectric layer, enables accurate pressure monitoring and patient comfort. The femtosecond laser (fsL)‐fabricated micropyramid arrays with stepped height ensures the linear relationship between the output signal of the capacitive sensor and the applied pressure, which allows the accuracy between the pressure display value and the measured value. Smart peripheral integrated reading circuits also ensure real‐time data transmission from the hemostasis device to monitoring mobile clients for medical assistance and direct visual observation. Unique advantages of the proposed wireless and compact sensing system are summarized in Note S1 (Supporting Information).

## 2 Results and Discussion

### 2.1 Engineering Mechanics of the Device

A hemostatic device is effective for preventing bleed to reduce the morbidity of vascular access complications. Radial compression device achieves the hemostasis by applying pressure on the radial artery (Figure 1a). To use the devices, 13–15 mL of air will be injected into the balloon according to the size of the wrist of patient. To quantitatively evaluate the applied pressure between air balloon and transradial, direct sensing and recording of the operation data provide more intuitive information and timely operation guidance. To ensure fast hemostasis and patient comfort, we assume a certain safe pressure range of 37.5–43 kPa. Figure 1b outlines the overall device layout, with images that demonstrate assembling components wearing on the wrist in Figure 1c. The design incorporates an all‐soft pressure sensor based on an LM electrode, peripheral electronic components, and adhesive sheets. EGaIn, a kind of room temperature LM, is an ideal soft electrode for application in soft electronics.^[^ ^28^, ^29^, ^30^, ^31^, ^32^, ^33^ ^]^ As shown in Figure S1 (Supporting information), the electronic part consists, more specifically, of a thin printed circuit board (PCB) based on an 80 µm thick middle polyimide supporting layer with 18 µm thick patterned copper (Cu) traces on the top and bottom surfaces. Immersion gold is used upon annealed Cu for more reliable solderability in the extra process. The electronic subsystems mainly include 1) a customized readout circuit for precise resistance sensing, with strong anti‐interference ability; 2) a microcontroller (MCU, nRF 52 832) for acquiring data from the reading circuit and communicating the results wirelessly via Bluetooth Low Energy protocols, and 3) a reserved power supply interface for optional rechargeable 150 mAh lithium‐ion polymer battery with programmed wireless charging function. As these subsystems highly rely on rigid, planar off‐the‐shelf components, they should be subtly integrated in a manner that simultaneously offers soft, shape‐compatible mechanics as well as effective data collection. The schemes used here exploit advanced versions of design concepts in stretchable electronics, adapted for use with the flexible PCB (fPCB) generally. Special interconnects coated by PDMS mechanically and electrically joint the PCB island of 20 × 40 mm^2^ and sensor island of 5 × 5 mm^2^.

The pressure sensor sticking on the air balloon directly contacts with the wrist for more accurate detection of applied pressure. LM‐based sensors can fully adapt with the deformation of the air balloon, and can maintain high sensing performance under large deformation. Meanwhile, the all‐soft sensor also ensures patient comfort when in contact with the skin and avoids secondary injury. A reference voltage module is combined with the low‐dropout regulator (LDO)  for precise capacitance measurements (Figure 1d). All the tested data would be processed and presented on a compact user interface for revealing real‐time applied pressure parameters and possible operation alerts (Note S2, Supporting information). These results highlight the customized sensors, subtly integrated circuits, and reasonably designed layouts necessary to accommodate realistic user requirements with stable and reliable sensing ability.

### 2.2 Design and Characterization of All‐Soft Sensors

**Figure 2:**
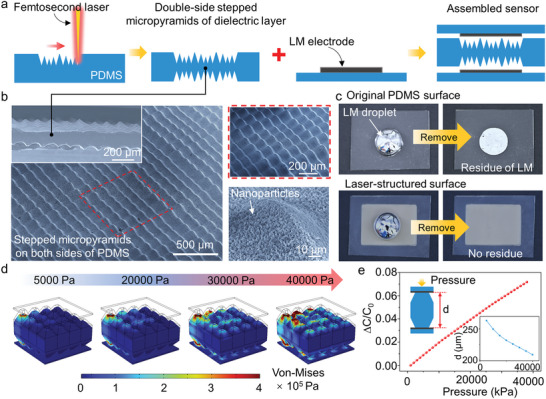
Design and characterization of the all‐soft pressure sensor. a) FsL fabrication of the dielectric layer with double‐sided stepped micropyramids for all‐soft capacitive sensor. b) SEM images of the dielectric layer with double‐sided stepped micropyramids. c) Adhesion between the LM and the original PDMS surface, and the laser‐structured surface, respectively. d) Stress distribution of simulation results for stepped micropyramids under pressures up to 40 kPa. e) Linear simulated result of the capacitance response to pressure from 0–40 kPa.

The microstructured dielectric layer has been introduced to improve the sensing performance of the capacitive pressure sensor.^[^ ^34^, ^35^, ^36^ ^]^ The sensitivity of the capacitive sensor can be defined as the slope of the curve (∆C/*C*_0_)/∆P, where C is the capacitance of the pressure sensor, *C*_0_ is the initial capacitance of the pressure sensor, P is the pressure applied to the pressure sensor. The ∆*C* of the sensor is contributed to the change in the distance (*d*) and the dielectric constant (*ε*) between the two electrodes. The microstructured dielectric layer allows far less resistance to applied pressure and more increase of *ε* because there are more air voids (*ε* = 1) replaced by PDMS (*ε* ≈2.7). FsL has the capability of cold machining due to its ultra‐short pulse and ultra‐high peak power and has significant application value in the field of micromaching.^[^ ^37^, ^38^, ^39^, ^40^, ^41^, ^42^, ^43^, ^44^ ^]^ The advantages of the FsL fabrication for microstructures are analyzed in Note S3 (Supporting information). **Figure** **2**a shows the fabrication process of the LM‐based capacitive pressure sensor. The preparation of the all‐soft pressure sensor is as follows: 1) the stepped micropyramid arrays are constructed on the surface of the PDMS substrate by an FsL; 2) turning over the surface of the PDMS and ablating the PDMS surface in the same way, a dielectric layer with double‐side micropyramids is prepared; 3) soft electrodes is prepared by directly printing the LM on the surface of the PDMS substrate; and 4) the capacitive pressure sensor is assembled by oxygen plasma bonding of the dielectric layer and the soft electrodes. Enabled by the high‐precision processing and superior controllability, fsL micromachining technology offers a route for facile and streamlined fabrication of dielectric layer with double‐side stepped micro‐pyramid arrays (Figure 2b; Figures S2 and S3, Supporting Information). The fsL processing is accompanied by the generating of the nanoparticles, so the hierarchical structures are formed on the surface of the dielectric layer, which repels the LM. As shown in Figure 2c and Figure S4 (Supporting information), an LM droplet is first dropped onto the original PDMS surface and the laser‐treated PDMS surface, respectively, and then the LM droplet is removed with a needle tube. A layer of highly adhesive oxide (Ga_2_O_3_) is formed on the outer surface of the LM, which can adhere to the surface of the untreated PDMS, allowing the printing of the LM‐based soft electrode. Conversely, fsL‐treated surfaces with hierarchically rough structures exhibit non‐sticky properties to LM, which allows LM to directly contact the dielectric layer with a micro‐pyramid structure without damaging the electrode. The property of the dielectric layer that repelling to LM ensures the robustness of the LM electrode of the capacitive sensor.^[^ ^32^ ^]^

To realize the accurate detection of the applied pressure on the skin, the linear sensitivity of the sensor is necessary. The micropyramid array was introduced to improve the sensitivity of the LM‐based pressure sensor, and the stepped micropyramids were further designed to achieve linear sensitivity to applied pressure. The sensitivity can be defined as S = (△C/C)/△P, where S is the sensitivity of the sensor, *C* is the capacitance of the sensor, and P is the value of the applied pressure. The C can be calculated by the following formula: *C* = *ε*A/*d*, where *ε* is the dielectric constant, A and *d* is the area of the electrode, and the distance between the two electrodes, respectively. The microstructure in the dielectric layer will produce air voids, which makes the far less elastic resistance to the applied pressure in the dielectric layer. When the structured dielectric layer is compressed, and air voids (*ε* = 1.0) will be displaced by the PDMS (*ε* = 2.7), resulting in an increase in capacitance value. However, the elastic resistance in the dielectric layer will increase sharply with the increase of the applied pressure due to the hyperelastic properties of the PDMS. Therefore, the conventional uniform microstructure will have a matter that the sensitivity of sensor will decrease sharply with the increase of the applied pressure so that the capacitance signal has a nonlinear response to the applied pressure. The elastic resistance in the dielectric layer can further be tuned by adjusting the specific shape and arrangement of the microstructures, thereby modifying the issue of nonlinear response of the capacitance signal to applied pressure. Figure 2d shows the simulation results of finite element analysis (FEA) and the stress distributions of the dielectric layer with stepped micropyramids during the gradual compression. The stress is mainly concentrated on the partial microstructure at the beginning of the compression due to the stepped microstructure, and the elastic resistance is smaller than that of the uniform microstructure. As the pressure gradually increases, the stress inside the dielectric layer gradually increases, so there will be no sudden increase in elastic resistance. As shown in Figure 2e, the simulated output capacitance shows a linear response to applied pressure. The distance between the two electrodes decreases linearly with the increase of pressure.

**Figure 3:**
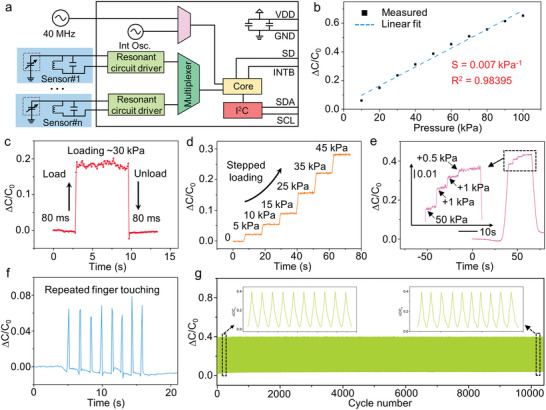
Sensing performance of the all‐soft sensor. a) Single amplifier reading circuits. b) Linear sensitivity (0.007 kPa^‐1^) with a linear correlation coefficient of 0.98395 over a wide pressure range up to 100 kPa of the sensor. c) Instant response time (<80 ms) of the sensor. d) Capacitance response to stepped loading of the sensor. e) Detection of micro pressure under loading pressures of 50 kPa. f) Transient response to the finger‐touching process. g) Stability of the sensor under repeated pressure (50 kPa) over 10 000 cycles.

Converting capacitive signal to digital value can be realized by many reading circuits, benefited by the input high‐frequency stimuli or charging current.^[^ ^8^ ^]^ The collected capacitance signals are passed to a resonant circuit driver, followed by an additional multiplexer with high sampling rates, demonstrated in **Figure** **3**a. To minimize the required PCB components with a smaller occupied area, I^2^C communication method is applied connecting to MCU parts. Linearity of the signal and the sensing range are the key parameters of the sensor in this application. Our designed sensor exhibits exceptional linear response to applied pressure over a wide range up to 100 kPa (Figure 3b). This result agrees well with the expectations we want to achieve by designing the dielectric layer with double‐side stepped micropyramids. The linear response to pressure is significant because it allows to provide users with accurate information even at high‐pressure range without the need for additional signal processing. The sensor shows a fast response time of lower than 80 ms, indicating that it has a good dynamic response capability and can respond quickly to pressure changes (Figure 3c). Introducing microstructure can accelerate the response speed of the sensor due to that the microstructure can quickly store and release energy for elastic recovery. As shown in Figure 3d, when the load increases linearly, the sensor output a linearly changed capacitance. It shows that when the sensor is used for monitoring the pressure, the pressure signal can be accurately output. In the application of the radial artery pressure monitoring, it is necessary to adjust the injected air in the air balloon according to the bleeding of the patient's puncture port. Therefore, it is significant for the sensor to be able to identify the variation of the tiny pressure when a certain pressure has been loaded. The sensor shows the ultra‐low limit of detection of ≈1.11 Pa (Figure S5, Supporting Information). As shown in Figure 3e, when the pressure of 50 kPa is first loaded on the surface of the sensor, and then the pressure of 1 and 0.5 kPa is gradually loaded, the sensor can still detect the tiny pressure change and make the signal change response. It shows that the sensor has the capability to identify small pressure when large pressure has been loaded. We use the finger to repeatedly press the surface of the sensor and see that the sensor can still stably output the capacitance change signal, indicating that the capacitive couples of the human body to the sensor can be almost ignored due to the enhancement of the sensitivity of the sensor by laser structured dielectric layer wit double‐side micropyramids (Figure 3f; Figure S6, Supporting Information). In order to verify the dynamic stability of the sensor, we repeatedly loaded 10 000 times with a force of ≈50 kPa (Figure 3g). The sensor shows a very stable signal output, indicating that it has outstanding stability and meets the needs of long‐term use. All these excellent performances indicate that the sensor can be used as a real‐time pressure monitoring device to assist hemostasis during postoperative surgery.

### 2.3 Sensing Performance of Strain Deformation and Applications

**Figure 4:**
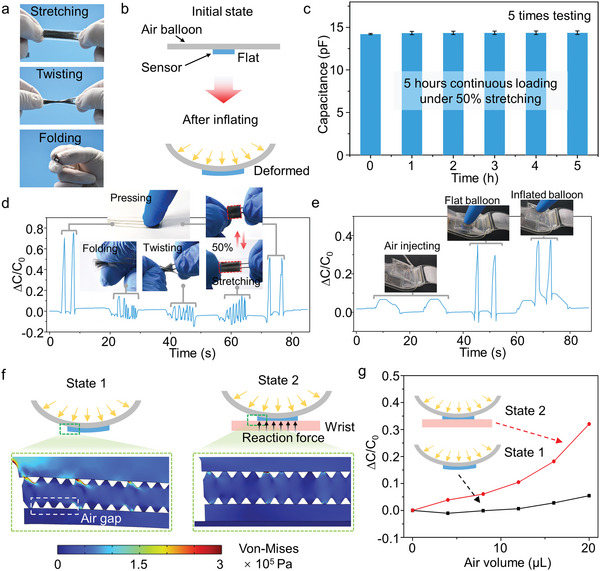
Insensitivity of the sensor to stretching and inflating deformations. a) Demonstration of stretchability of the sensor. b) Schematic demonstration of deformation of the sensor during the inflating of the air balloon. c) Stability of the sensor under 50% tensile strain over 5 h. Demonstration of sensor insensitivity to d) deformations of bending, twisting, and stretching, and e) inflating. f) Stress distribution of simulation results for sensor under state 1 (only inflating) and state 2 (inflating while wearing on the wrist), g) capacitance response of the sensor to state 1 and state 2.

The use of intrinsically soft electrodes with near zero Young's modulus provides adaptability to elastic deformations, such as 100% stretching, twisting, and folding, as shown in **Figure** **4**a. The key advantages of the LM‐based all‐soft pressure sensor introduced here lie in its outstanding compliance with the deformed air balloon, and stable sensing under large tensile strain (Figure 4b). Since the sensor attached to the air balloon of the hemostasis device will be subjected to tensile deformation during the air balloon inflation process, it is necessary for the sensor to be insensitive to tensile deformation and still have excellent pressure detection capability under tensile conditions. The sensor shows stable sensing performance under 50% strain tensile during the 5 h of continuous loading (Figure 4c), and dynamic stretch‐release cyclic loading (Figure S7, Supporting Information), which shows excellent long‐time sensing stability under deformations. We found in the test that the deformation of the air balloon is ≈10% during the process from the uninflated state to the full expansion (Figure S8, Supporting Information). To demonstrate the stretchability and deformation‐unperturbed performance of our sensor, we test the capacitance response to elastic deformation and external pressure, respectively. Figure 4d and Movie S1 (Supporting information) show that the capacitance change caused by pressure is much larger than that caused by elastic deformation of 50% stretching, twisting, and folding. Furthermore, we attach the sensor to air balloon of the hemostasis device to demonstrate the strain‐insensitivity of the sensor in application of the pressure monitoring (Figure 4e).

To elucidate the underlying mechanism by which the soft sensor with a stepped micropyramids dielectric layer shows strain‐insensitivity, we investigate the soft sensor attached to the air balloon without and with pressure applied by the wrist by performing FEA, respectively (Figure 4f; Figure S9, supporting information). When the sensor is deformed with the inflation of the air balloon, the dielectric layer is not significantly compressed, and only the relative position changes between the two electrodes. But when the pressure from the wrist is applied on the surface of the sensor, the stepped micropyramids of the dielectric layer will be totally compressed, which contributes to the remarkable increases of the output capacitance signal. The FEA result of the insensitivity to tensile deformation is also demonstrated in Figure S10 (Supporting Information). Figure 4g shows the signal output of the sensor without wearing on the wrist (state 1) and after wearing on the wrist (state 2) during the continuous inflation of the airbag respectively. It is further demonstrated that in clinical applications, the deformation of the sensor caused by the inflation of an air balloon shows negligible interference on the signal of the sensor. All the above results can prove that the signal output by the sensor can effectively reflect the actual pressure applied on the puncture wound by airbag of the hemostasis device.

**Figure 5:**
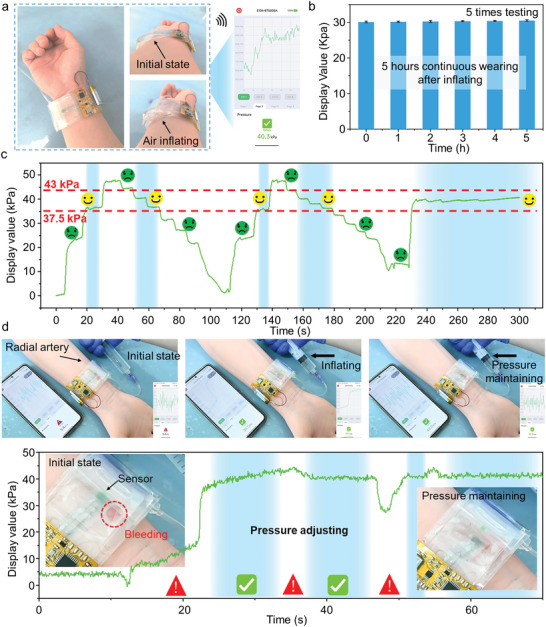
Demonstration of the application of smart hemostasis devices. a) Smart hemostasis devices worn on the wrist. b) Long‐time stability of the sensor wearing on the wrist over 5 h with the air balloon inflating. c) Safe hemostasis pressure range during the clinical surgery. d) Demonstration of using smart hemostasis devices to monitor the hemostasis pressure in real‐time.

To test the functionality of our hemostasis device in clinical surgery, we performed a series of experiments to verify its usability. Due to the combination of the compact and wireless processing circuit and the all‐soft sensor, the hemostasis device integrated with the sensing system will not cause inconvenience to the operation and discomfort to the human body when worn on the wrist (**Figure** **5**a). In order to test the long‐time stability of our sensing system, we carried out a 5 h constant pressure test on the wrist that wearing the hemostasis devices. The detected pressure applied on the radial artery remained constant after 5 h (Figure 5b). In clinical surgery, the applied pressure to the radial artery is allowed neither to be too large nor too small, so it is necessary to determine the safe pressure range. We take the standard that people can feel obvious pressure and will not cause discomfort due to excessive pressure to determine the safe pressure range (Figure 5c). After repeated pressure adjustment and observation of volunteer's feedback, the safe pressure was finally set at a range of 37.5–43 kPa (Figure S11 and Movie S2, Supporting information). As shown in Figure 5d and Movie S3 (Supporting information), we conducted a hemostatic test on a 1:1 silicone human arm (with simulated blood vessels inside) to confirm that the pressure range (37.5–43 kPa) determined here can play a good hemostatic effect. At the initial state, there will be massive blood exudation at the puncture port, but as the gas continues to fill the airbag and the pressure reaches a safe pressure range, the bleeding disappears. Through subsequent pressure adjustment, the pressure is within a stable range, and long‐term hemostasis can be performed. By quantitative and visual display of the pressure applied to the radial artery, our sensing system can provide more intuitive assistance to doctors. More importantly, for individual differences of the patients with different wrist sizes, accurate hemostatic pressure can also be adjusted, which can greatly reduce the operating risk because of the subjective judgment of doctors in clinical operations.

## 3 Conclusion

In this study, we have developed a compact and wireless pressure sensing system upon a hemostasis device to achieve real‐time pressure monitoring applied on the user's wrist. The system consists of capacitive pressure sensor, tiny reading circuits with low‐power transmission functions, and software‐based model deployment. The all‐soft pressure sensor based on LM is introduced for detecting the pressure under large deformation of the air balloon, and also ensures the wearing comfort of the patients. The construction of PDMS dielectric layer with double‐sided stepped micropyramids endows the sensor with exceptional linear response to pressure over the range of 0–00 kPa, and improves the accuracy of the data display. With our devices, detailed pressure data on retracting operations can be collected, analyzed, and stored for medical assistance as well as to improve surgery quality. The device can also determine the most appropriate pressure range and time during the hemostatic through repeated tests, and provide intuitive guidance for surgical hemostasis. In summary, this work provides broader applications not only for wearable electronics but also the intelligent medical care services.

## 4 Experimental Section

### Materials

The LM used in the experiment was EGaIn (Wochang Metal Co., Ltd.), which had a melting point of 12 °C. PDMS layers were purchased from Hangzhou Bald Advanced Materials Co., Ltd. The silicon human arm was purchased from BOLE TEACHING MODEL.

### Preparation of the Sensor: Fabrication of the Microstructures

Here, the stepped PDMS micropyramids of dielectric layer (300 µm in thickness) were structured by an FsL orthogonally crossed line‐by‐line ablation (Figure S12, Supporting information). A FsL beam (with a pulse duration of 50 fs, central wavelength of 800 nm, and repetition frequency of 1 kHz) from a Ti: sapphire laser system (Coherent, Librausp 1K‐he200) was vertically focused onto the surface of the PDMS sheet by a plano‐convex lens (focal length of 200 mm) in air. The PDMS substrate was fixed on a computer‐controlled moveable platform. The laser power was held constant at 300 mW and the moving speed of the platform was 50 µm s^−1^. Adjusting the adjacent distance (AD) of laser scanning could realize the preparation of stepped micropyramids (Figure S13, Supporting information).

### Preparation of the Sensor: Patterning the LM Electrode

First, the mask plate of the designed pattern was covered on the PDMS surface, and then the LM was poured onto the surface of PDMS substrate covered with the mask. Finally, the mask plate was slowly removed to form a patterned LM electrode on the surface of PDMS (Figure S14, Supporting information).

### Preparation of the Sensor: Assembly of the AFTS

The sensors were assembled by bonding the dielectric layer and PDMS supporting layer printed with LM electrode after oxygen plasma treatment. First, the copper electrode was stuck to the LM electrode of the PDMS support layer for subsequent testing, and then this surface was bonded to one side of the dielectric layer to form a stable electrode connection and encapsulation. Then, the copper electrode was connected to the LM electrode by repeating the previous step, and the support layer with LM was bonded to the other side of the dielectric layer to form the final sensor (Figure S15, Supporting Information). The prepared all‐soft sensor could withstand complex elastic deformation, such as stretching, twisting, and squeezing (Figure 4a).

### Characterization

The surface microstructures of the samples were observed by a Flex 1000 scanning electron microscope (SEM; Hitachi, Japan). The wettability of liquid EGaIn droplets and water droplets on the sample surface was investigated by a JC2000D contact angle (CA) system (Powereach, China). The 3D morphology of the surface of the stepped micropyramid arrays was characterized through a LEXT‐OLS4000 laser confocal microscope (Olympus, Japan). The capacitance was measured by a WK4100 LCR meter (Wayne Kerr Electronics, U.K.). All capacitance signals were tested at a frequency of 1 MHz.

### Fabrication of Electronics

The LDO was fabricated in SMIC 180 nm CMOS process, which occupies a chip area of 0.046 mm^2^, with the die microphotograph. In this study, the LDO could support an input voltage range of 0.8–3.3 V and an output voltage range of 0.6–3.2 V. The maximum load current of the LDO was 201 mA with an external Capacitance of 1 µF for testing purposes and no internal capacitor was implemented on‐chip. The average parameters of amplifier DC gain, unity‐gain bandwidth, phase margin, and slew rate were 94.18 dB, 4.81 MHz, 58.42°, and 2.53 V µs^−1^, respectively. Besides, the error amplifier consumes little quiescent current and the maximum current efficiency of the LDO was 99.99%. Other commercial chips were also assembled on a sheet of fPCB into compact layouts. Solder paste (Chip Quik TS391LT) and a heat gun (AOYUE Int866) joined the various electronics and sensors onto the fPCB.

### Statistical Analysis and Software Design

The participants were all recruited by the Xi'an Jiaotong University and entirely voluntary. For all the studies, the participants gave informed consent. The devices used were considered to carry minimal risk, and therefore approval was not needed. Collected data analysis was performed on MATLAB (R2018b) with technical computing language and Origin (2019b), and the initial quantitative analysis results of sensing parameters were obtained from five samples. The sensing model was converted into a lightweight version and deployed into Android on Android Studio (2021.1.1.23) with PyTorch Android lite library. The displayed mobile clients included a Redmi K50 (equipped with Android 12 system).

## Conflict of Interest

The authors declare no conflict of interest.

## Supporting information

Supporting Information

Supplemental Movie1

Supplemental Movie2

Supplemental Movie3

## Data Availability

The data that support the findings of this study are available in the supplementary material of this article.
