# Supplementary material for: Wireless, Smart Hemostasis Device with All‐Soft Sensing System for Quantitative and Real‐Time Pressure Evaluation
Source: Adv Sci (Weinh). 2023 Sep 8;10(33):2303418. doi: 10.1002/advs.202303418 (PMC10667811; doi:10.1002/advs.202303418)
Supplement: Supplementary file 1 — Supporting Information [file ADVS-10-2303418-s004.pdf]

ADVANCED SCIENCE
Open Access

Supporting Information

for *Adv. Sci.*, DOI 10.1002/advs.202303418

Wireless, Smart Hemostasis Device with All-Soft Sensing System for Quantitative and Real-Time Pressure Evaluation

Chengjun Zhang, Qing Yang, Xianglin Meng, Haoyu Li, Zexiang Luo, Lin Kai, Jie Liang, Sicheng Chen* and Feng Chen**

Supporting information

Wireless, Smart Hemostasis Device with All-Soft Sensing System for Quantitative and Real-Time Pressure Evaluation

Chengjun Zhang[1], Qing Yang[1,*], Xianglin Meng[4], Haoyu Li [2], Zexiang Luo[2], Lin Kai[2],Jie Liang[2], Sicheng Chen[3,*] and Feng Chen[2,*]

*Corresponding author. Email:
*chenfeng@mail.xjtu.edu.cn (Feng Chen)
*sicheng.chen@ntu.edu.sg (Sicheng Chen)
* yangqing@mail.xjtu.edu.cn (Qing Yang)

This PDF file includes:

Supplementary Text
Figures. S1 to S15
Movies S1 to S3

Other Supplementary Materials for this manuscript include the following:

Movies S1 to S3

Supplementary Text

Note S1. Unique Advantages for the Proposed Wireless and Compact Sensing System.

In this study, our proposed sensing system possesses wireless communicating functions, which would endow the hemostasis device with two specific superior advantages. 1) Portable medical instruments will not restrict the doctors' freedom while they are operating in complex environments, filled with various surgical machines. 2) Sensor data quality through wired transmission can be usually affected by surrounding electronic interference, especially for capacitive type sensors. Wireless data transmission would be more suitable for indoor sensing signal collection, as the data processing is finished in situ chip-level.

Note S2. System Operation Flow

Device operation: The user interface controls the device operation. To turn on the system, it first connects to the BLE Mac address of the device. By default, the device starts working after pressing the CONNECT button. A second pressing-on action would disconnect the device. The user interface starts and stops recording the data to an onboard flash memory within the device or uploading the data to cloud client if needed. When the session begins, the MCU receives the data from the reading circuit and writes it to the flash memory. During the recording session, users can

monitor the multi-index and real-time curve anywhere as long as an equipment is connected to the internet.

Power-flow: The power from Li-ion battery goes through the LDO (DC/DC converter) that regulates it to 3.3 V and delivers to the active components throughout the system, which includes a microcontroller, pressure sensor, reading IC and wireless communication circuits.

Note S3. Superiority of the femtosecond laser fabrication for microstructures

Femtosecond laser (fsL) technology is a flexible micro-nanofabricating method for constructing the micro/nanostructures in a low-cost, highly controllable, and rapid manner. [1-4] Various complex microstructures can be directly fabricated without expensive mask and complex technological process. The extremely high peak intensity associated with an ultrashort pulse width of fsL enables the cold processing of the transparent elastic materials (PDMS), and ensures the minimal damage to the non-ablated regions. Over the years, microstructures have been introduced to improve the sensing performance of the pressure sensor, especially capacitive sensor. [5-8] The microstructured dielectric layer endows the sensor with higher sensitivity, and faster response time. Here, we fabricated a dielectric layer with double-sided stepped micropyramids by a femtosecond laser microfabrication with a maskless fabrication. The double-sided micropyramids dielectric layer havs been proved the superiority of the sensing performance compared to the one-side microstructured dielectric layer fabricated by conventional method, such as photolithography, and transferring method. [9] The arrangement and morphology of the microstructure can be effectively tuned by adjusting the parameters of laser processing (laser power, scanning speed, and adjacent scanning distance), which allows the optimal adjustment of the sensing performance.

References:

[1] K. Sugioka, *Int. J. Extrem. Manuf.* **2019**, *1*, 012003.

[2] X. Bai, Q Yang, Y. Fang, J. Zhang, J. Yong, X. Hou, F. Chen, *Chem. Eng. J.* **2020**, *383*, 123143.

[3] J. Yong, F. Chen, Y. Fang, J. Huo, Q. Yang, J. Zhang, H. Bian, X. Hou, *ACS Appl. Mater. Interfaces* **2017**, *9*, 39863.

[4] B. Xu, Y. Zhang, H. Xia, W. Dong, H. Ding H. Sun, *Lab Chip*, **2013**, *13*, 1677.

[5] S. C. Mannsfeld, B. C. Tee, R. M. Stoltenberg, C. V. Chen, S. Barman, B. V. Muir, A. N. Sokolov, C. Reese, Z. Bao, *Nat. Mater*. **2010**, *9*, 859.

[6] Z. Li, S. Zhang, Y. Chen, H. Ling, L. Zhao, G. Luo, X. Wang, M. C. Hartel, H. Liu, Y. Xue, R. Haghniaz, K. Lee, W. Sun, H. Kim, J. Lee, Y. Zhao, Y. Zhao, S. Emaminejad, S. Ahadian, N. Ashammakhi, M. R. Dokmeci, Z. Jiang, A. Khademhosseini, *Adv. Funct. Mater.* **2020**, *30*, 2003601.

[7] Y. Lee, J. Park, S. Cho, Y. E. Shin, H. Lee, J. Kim, J. Myoung, S. Cho, S.Kang, C. Baig, H. Ko, *ACS Nano* **2018**, *12*, 4045.

[8] G. Y. Bae, S. W. Pak, D. Kim, G. Lee, D. H. Kim, Y. Chung, K. Cho, *Adv. Mater.* **2016**, *28*, 5300.

[9] C. Zhang, Z. Li, H. Li, Q. Yang, H. Wang, C. Shan, J. Zhang, X. Hou, F. Chen, *ACS Appl. Mater. Interfaces* **2022**, *14*, 38328.

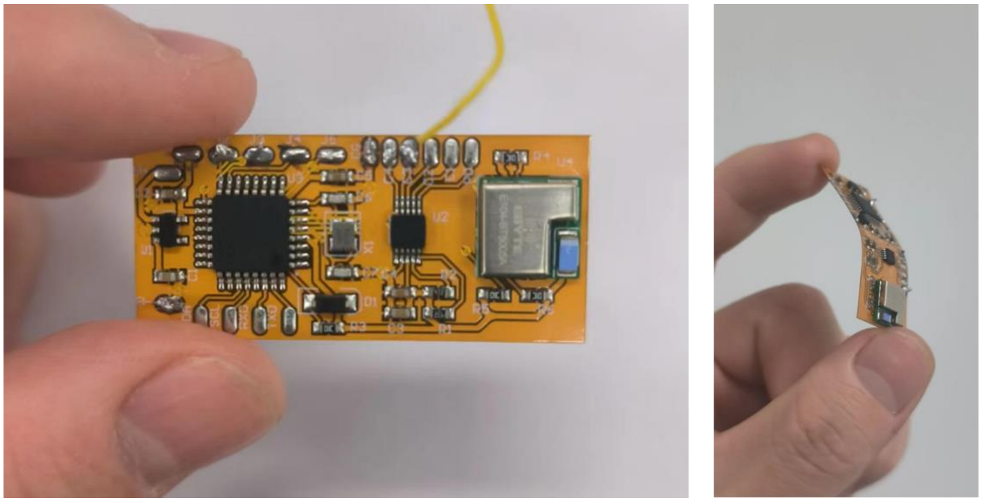

Figure S1. Images of the reading circuit.

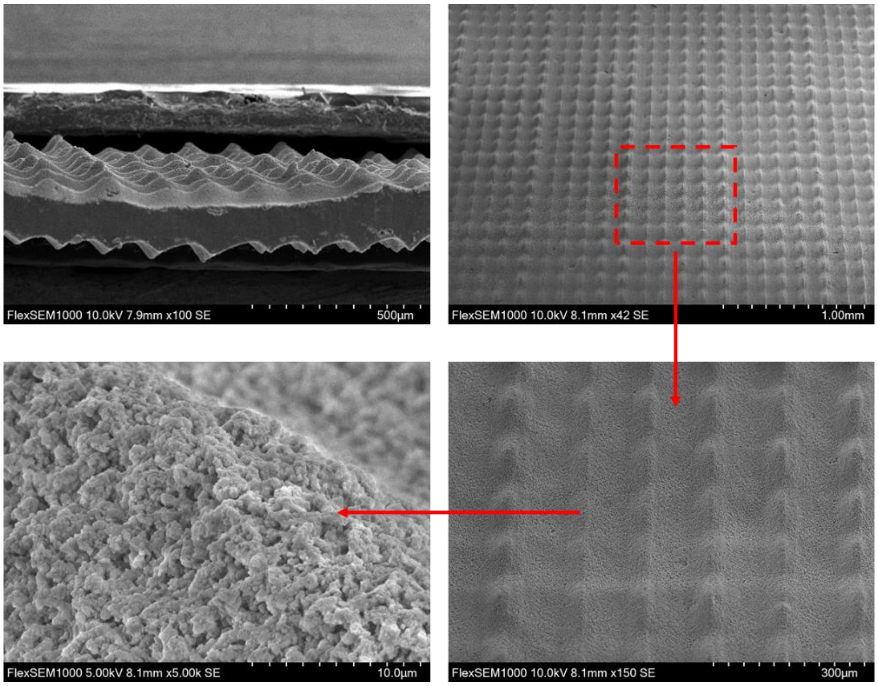

Figure S2. SEM images of the double-sided dielectric layer with stepped micropyramids.

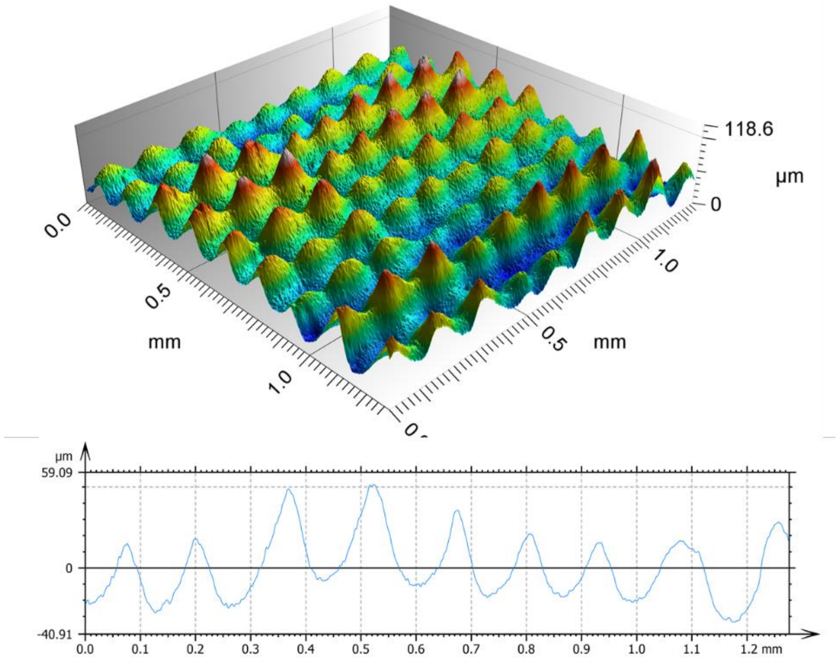

Figure S3. Three-dimensional (3D) morphology of the stepped micropyramids of the dielectric layer.

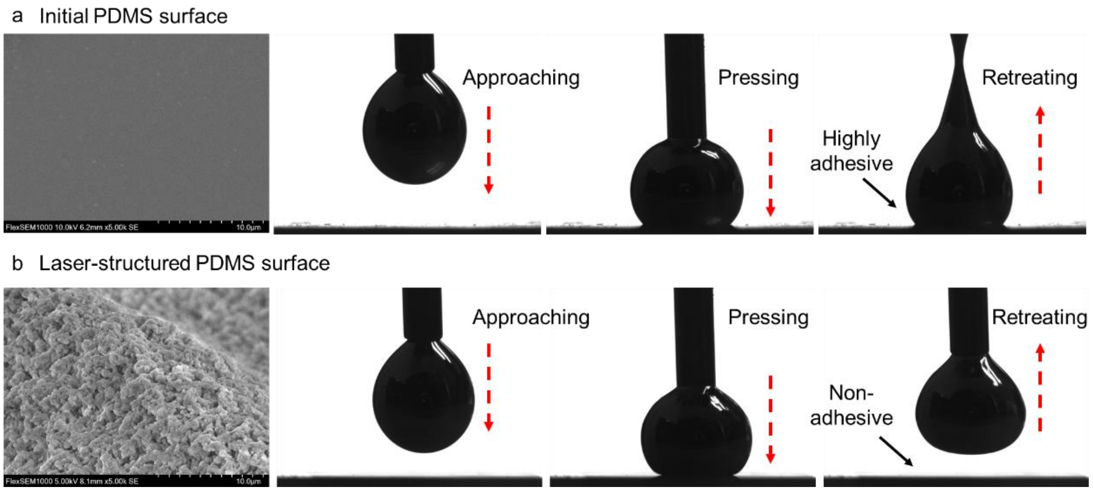

Figure S4. Wettability between the LM and (a) initial PDMS surface, (b) laser-structured PDMS surface.

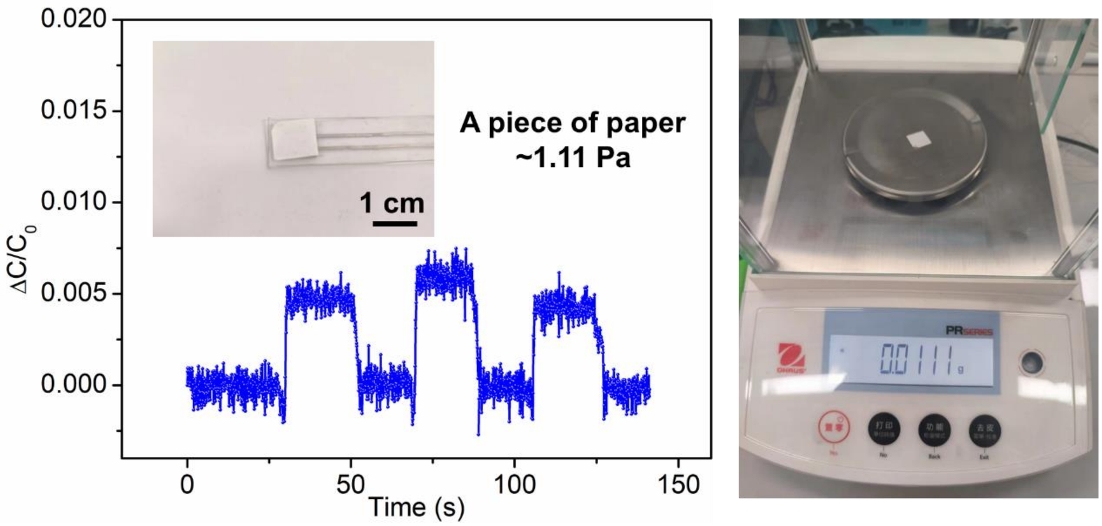

Figure S5. Limit of detection of the sensor.

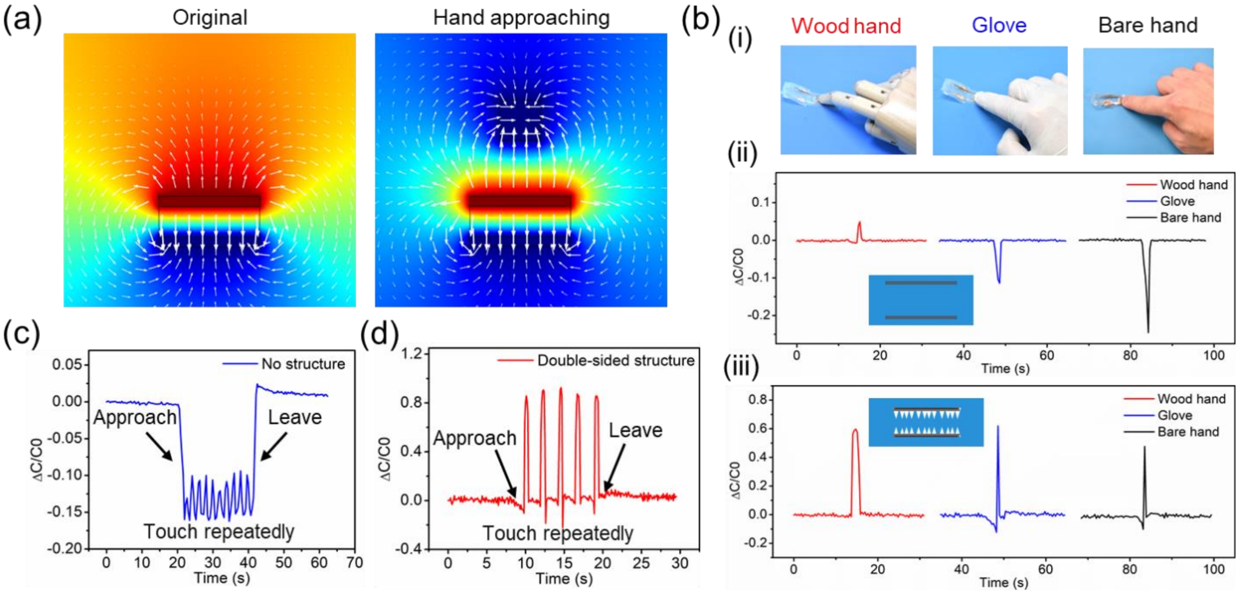

Figure S6. (a) Simulation results of human body charge for sensor electric field. (b) Optical images of external pressure applied on the sensor (approach, touch slightly, and leave) by wood, glove, and bare hand, respectively (i). Relative change of capacitance when external pressure applied on the sensor (by a wood, glove, and bare hand, respectively) (ii) before and (iii) after introducing the micropyramid arrays. Relative change of capacitance when an external pressure applied by a finger (approach, touch repeatedly, and leave) on the sensor (e) before and (f) after introducing the micropyramid arrays.

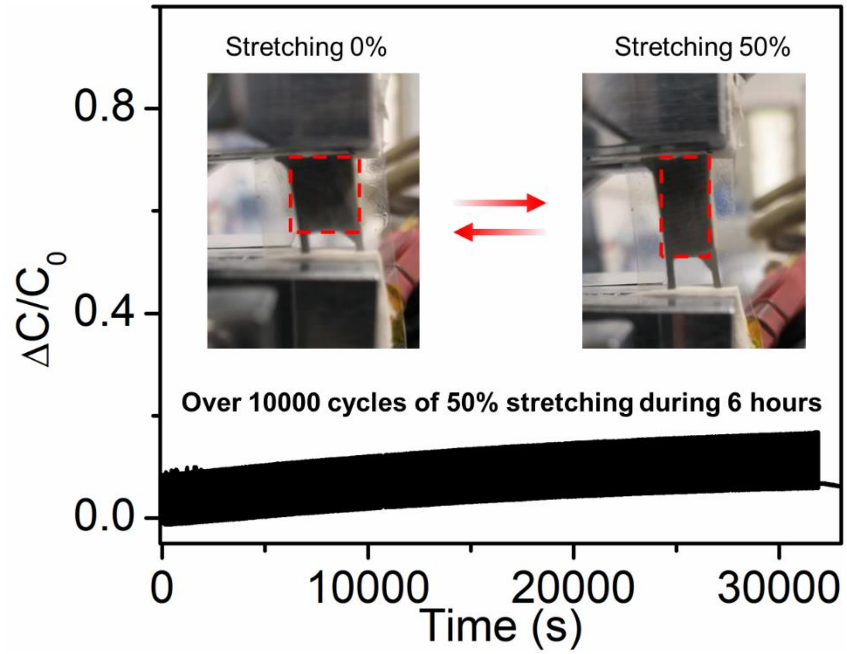
Stretching 0%
Stretching 50%
Over 10000 cycles of 50% stretching during 6 hours
ΔC/C0
0.8
0.4
0.0
0
10000
20000
30000
Time (s)

Figure S7. Dynamic cyclic stretching stability of the sensor over 10000 cycles.

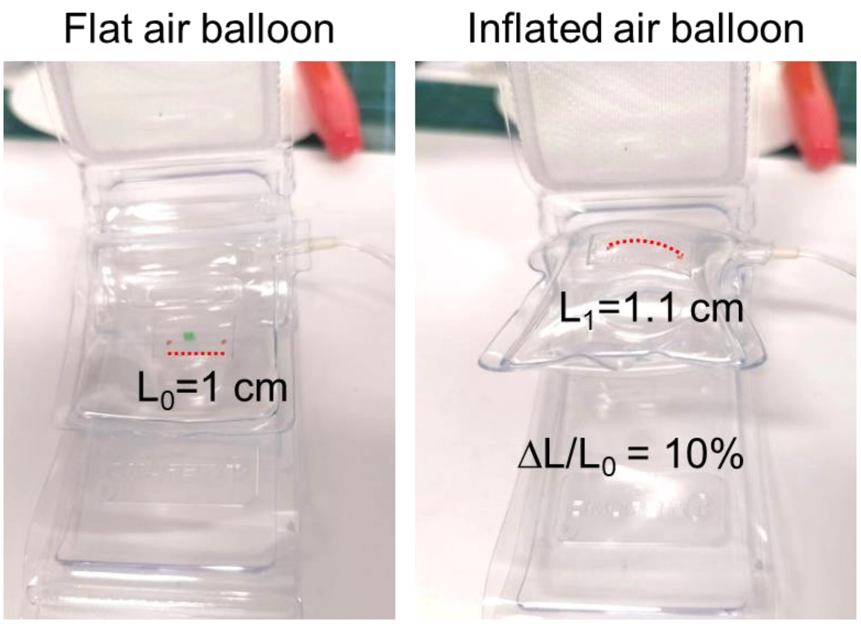

Figure S8. Distance changes of two points on the air balloon during the inflation.

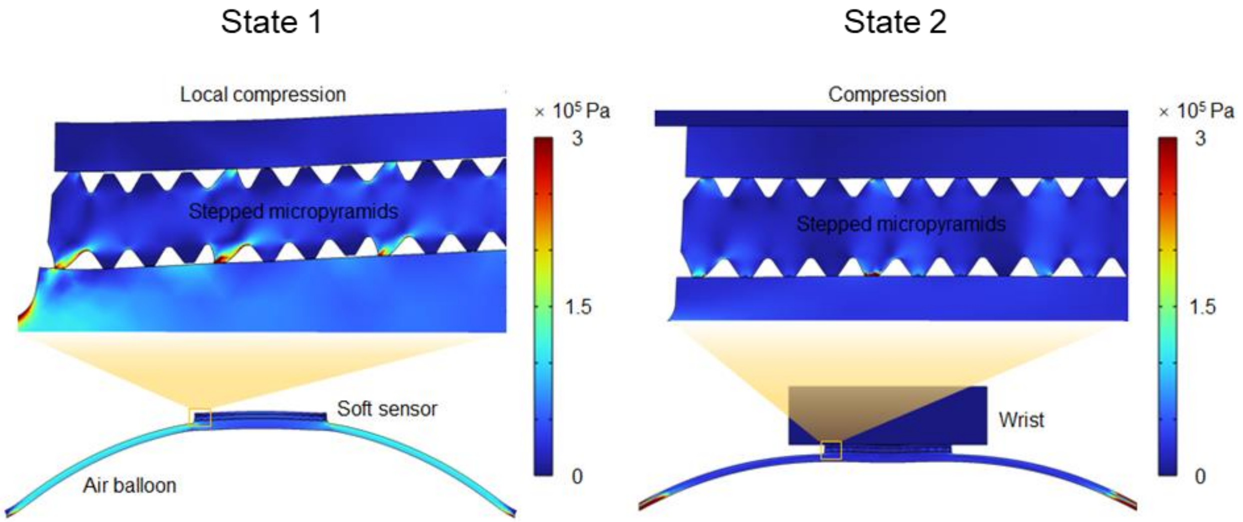

Figure S9. Stress distribution of simulation results for the sensor under state 1 (only air balloon inflating) and state 2 (air balloon inflating while wearing on the wrist).

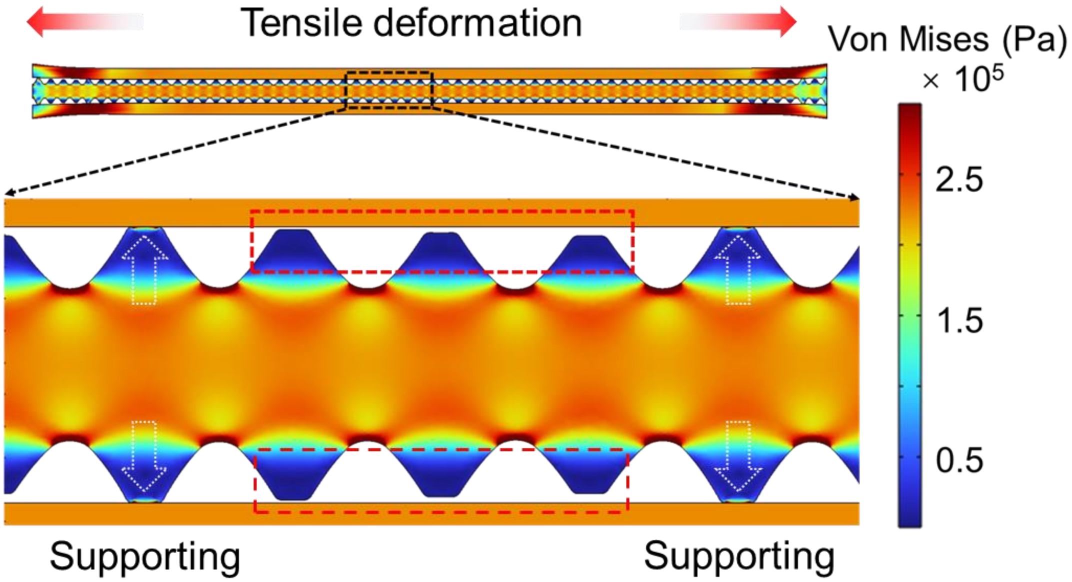

Figure S10. Simulation results of the sensor under tensile deformations. The micropyramids protruding on both sides of the intermediate dielectric layer with stepped microstructure are equivalent to two supporting columns. When the sensor is deformed by stretching, the supporting columns will bear the extrusion caused by stretching, and the microstructure between the two supporting columns will not be compressed. Therefore, capacitance change will also generate under tensile strain, but the signal intensity is much smaller than that generated by the vertical pressure.

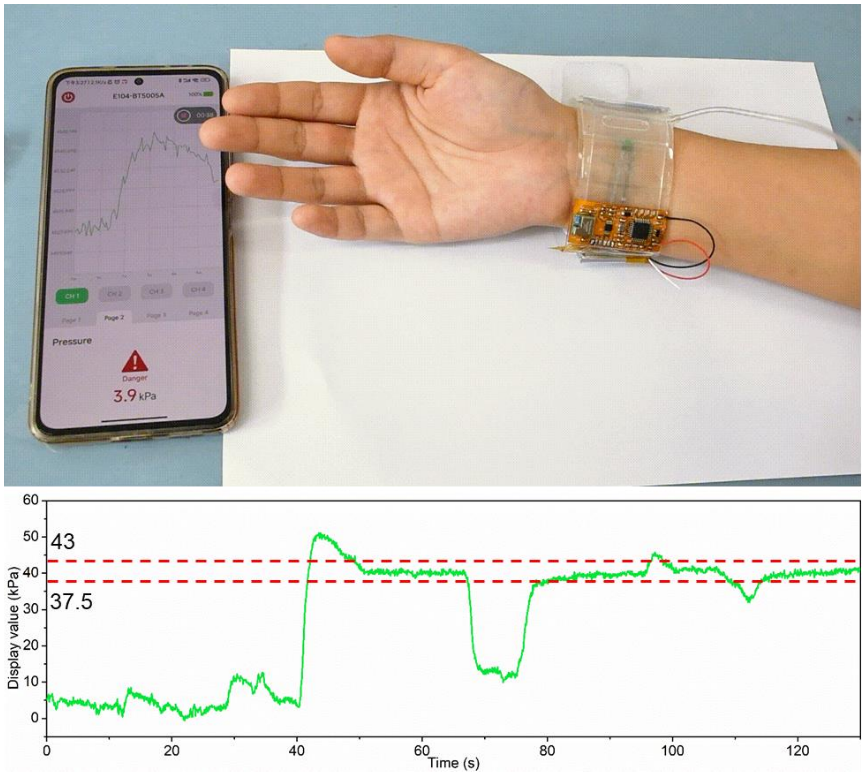

Figure S11. Demonstration of the pressure monitoring when the smart hemostasis devices is wearing on the human wrist.

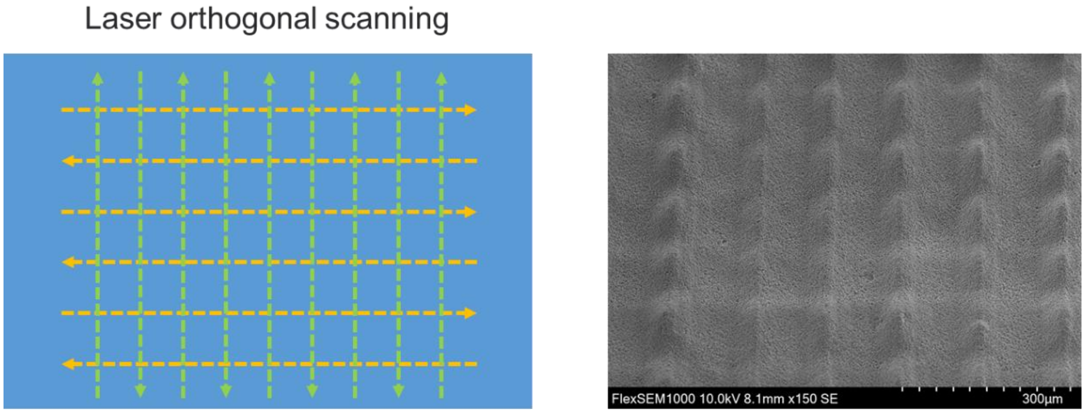

Figure S12. Fabrication of the micropyramids of the dielectric layer by femtosecond laser orthogonal scanning.

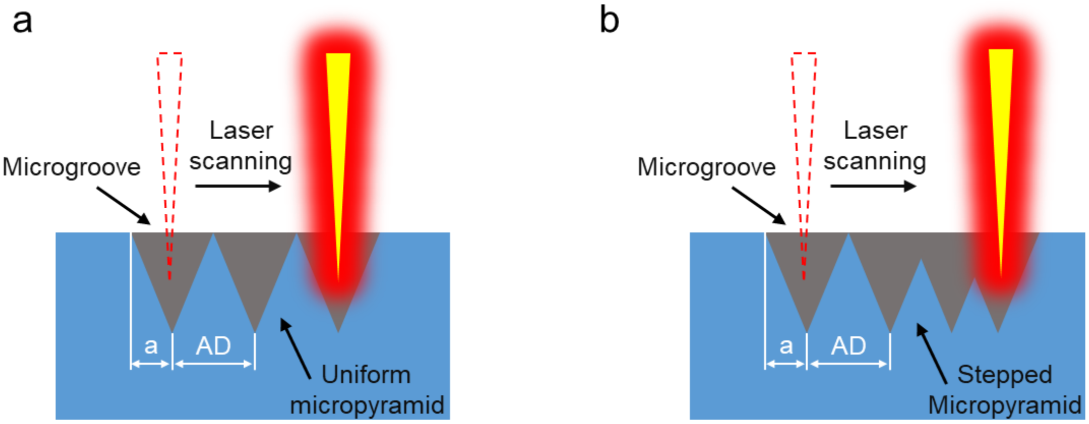

Figure S13. Laser scanning method to fabricate (a) uniform micropyramids, and (b) stepped micropyramids.

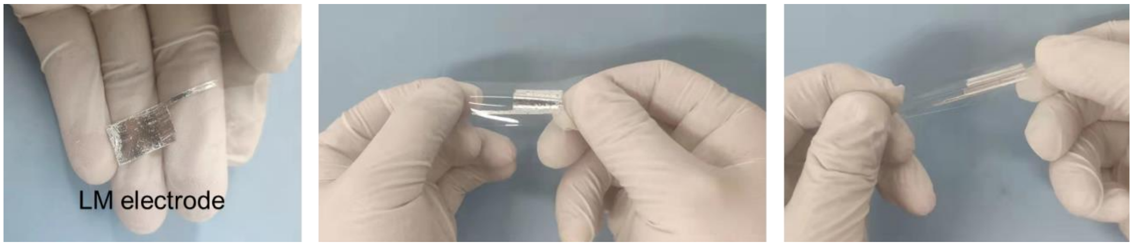

Figure S14. Printed LM electrode under stretching.

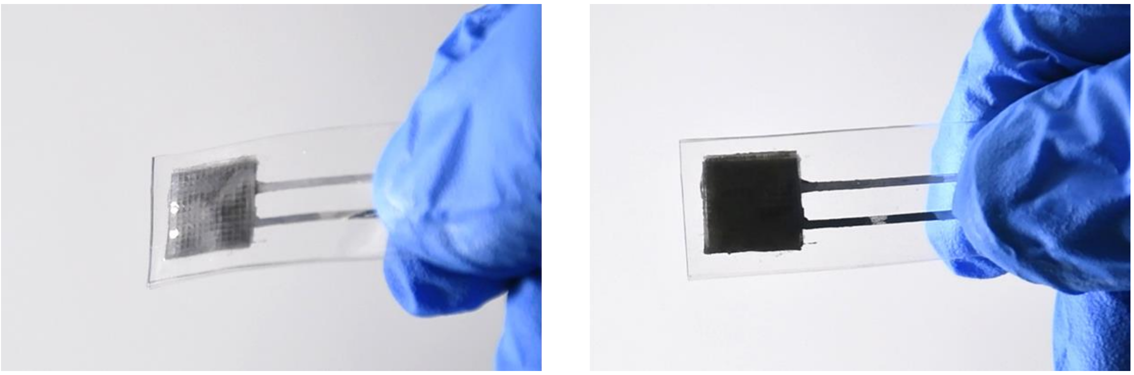

Figure S15. Optical images of the all-soft sensor.

Movie S1.

Demonstration of the insensitive performance to stretching deformation of the all-soft sensor.

Movie S2.

Pressure monitoring upon the wrist of the human.

Movie S3.

Application demonstration of the smart hemostasis devices.
